# Endonasal flap reconstruction in sinonasal malignancy

**DOI:** 10.3389/fonc.2026.1777655

**Published:** 2026-03-06

**Authors:** Kalpesh Hathi, Christopher J. Chin

**Affiliations:** Division of Otolaryngology – Head & Neck Surgery, Department of Surgery, Dalhousie University, Halifax, NS, Canada

**Keywords:** malignancy, nasoseptal, reconstruction, sinonasal, skull base, sphenopalatine

## Abstract

Sinonasal malignancies are a broad, yet rare, class of head and neck cancers with a poor prognosis. Surgical resection is the mainstay of treatment for the majority of tumors. Resection of sinonasal malignancies may result in cerebrospinal fluid (CSF) leak, meningitis, pneumocephalus, and prolonged nasal crusting if not appropriately reconstructed. The advent of endoscopic sinus surgery (ESS) has transformed the field and allowed for fully endonasal resection of sinonasal malignancies. The Hadad-Bassagasteguy flap, now colloquially known as the nasoseptal flap, has revolutionized endonasal reconstruction. The nasoseptal flap is a robust mucoperichondrial flap pedicled on the posterior septal artery, providing a rich and reliable blood supply. The nasoseptal flap has become the workhorse of anterior skull base reconstruction given its relative ease of harvest, reliability, low donor site morbidity and success: CSF leaks rates have decreased from > 20% to < 5% with the use of the nasoseptal flap. This review thoroughly discusses the history, use, and techniques for the nasoseptal flap.

## Introduction

Sinonasal malignancies are a broad, yet relatively rare, class of head and neck cancer. Accounting for less than 5% of head and neck malignancies, sinonasal malignancies impact less than 1 per 100,000 people (1, 2). Squamous cell carcinoma (SCC) is the most common sinonasal malignancy; other common pathologies include adenocarcinoma, B-cell lymphoma, epithelial neoplasms and melanoma (1, 2).

Despite its overall rarity, sinonasal malignancies are aggressive and have a poor survival, making the advancement of their treatment paramount. The 5-year disease-specific survival for sinonasal malignancies ranges from 30-70% (1, 3). Sinonasal malignancies most commonly arise from the nasal cavity but also routinely involve the paranasal sinuses (1, 4). Symptomatology with sinonasal malignancies may be subtle and result in presentation with advanced disease, contributing to poor overall outcomes. However, in resectable sinonasal malignancies, surgery remains the mainstay of treatment (4). Given the proximity of the nasal cavity and paranasal sinuses to vital neurovascular structures, surgical resection necessitates a deliberate approach to reconstructive planning. Resection of sinonasal malignancies can result in cerebrospinal fluid (CSF) leaks, meningitis, pneumocephalus, orbital complications or prolonged nasal crusting. Overall, this impacts patients’ quality of life, and ideal reconstruction can minimize its impact on patients.

Reconstruction of these resultant defects focuses on repairing CSF leaks, maintaining the nasal airway, restoring the barrier to intracranial contents and restoring sinonasal function. Reconstruction can range from local to free tissue transfer, depending on the extent of the resection. This article is a thorough narrative review of endonasal local flap reconstruction following resection of sinonasal malignancies. This narrative review provides an in-depth and up-to-date understanding of the history, technique, advantages and potential complications associated with endonasal flaps, with a focus on the nasoseptal flap. These well-established techniques are discussed in depth and effectively reviewed providing a concise resource on the topic.

## Historical perspectives

Historically, sinonasal malignancies were resected via open approaches. However, the advent of endoscopic sinus surgery (ESS) in the 1980s has reduced the need for open surgery. ESS is associated with improved quality of life compared to open approaches (5).

The field of endoscopic anterior skull base surgery was initiated with transsphenoidal pituitary surgery. Transsphenoidal surgery replaced craniotomy for pituitary lesions, which historically had high rates of mortality (6, 7). Transsphenoidal surgery has developed from microscopic to now more commonly, endoscopic surgery. Despite these advances in ESS, resultant skull base defects were typically reconstructed using onlay grafts, which had > 10-20% CSF leak rates (7, 8). At that time, pedicled vascularized flaps still required extranasal approaches using pericraneal, galeal, or temporoparietal flaps (7). This approach was revolutionized by the advent of the nasoseptal flap.

## Nasoseptal flap

The nasoseptal flap was first described by Hadad et al. in 2006 and was initially named the Hadad-Bassagasteguy flap (9). Stemming from the original 43-patient case series in 2006, the Hadad-Bassagasteguy flap gained popularity and is often referred to now as the nasoseptal flap. This flap is now considered the main workhorse in anterior skull base reconstruction (9). The benefits of the nasoseptal flap are an entirely endonasal/endoscopic approach, reducing patient morbidity, while providing a robust vascularized tissue reconstruction.

In the initial 2006 article, only 2/43 patients had persistent CSF leaks and further literature has shown the nasoseptal flap to reduce the rate of CSF leaks from 12.5 to 3.2% (8, 9).

### Anatomy

**Figure 1:**
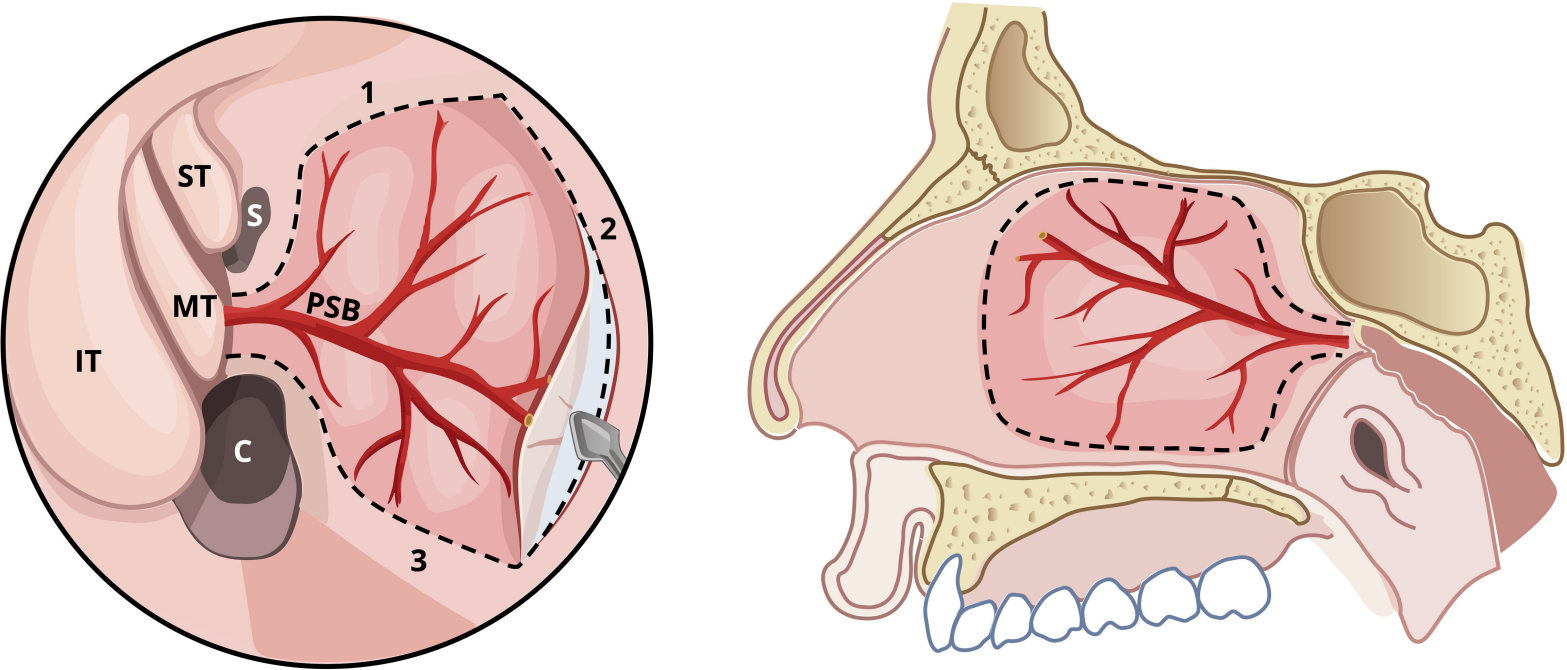
Illustration of nasoseptal flap incisions, plane of harvest and anatomy of sphenopalatine artery. S, Sphenoid sinus; C, Choana; ST, Superior Turbinate; MT, Middle Turbinate; IT, Inferior Turbinate; PSB, Posterior septal branch of sphenopalatine artery.

The nasoseptal flap is pedicled on one of the terminal branches of the sphenopalatine artery (SPA), specifically the posterior septal branch, allowing for a robust and reliable vascular supply with a wide arc of rotation (7, 9, 10). The SPA enters the nasal cavity via the sphenopalatine foramen as a terminal branch of the internal maxillary artery from the external carotid (7, 9, 10). The anatomy of the SPA is visualized in **Figure 1**.

### Pre-operative preparation

Pre-operative preparation begins with ensuring thorough endoscopic visualization as well as CT imaging of the patient’s sinonasal anatomy. Following induction of general anesthesia, nasal pledgets with topical decongestants (topical epinephrine or oxymetazoline) are placed in the nasal cavity to reduce edema and inflammation, improving visualization (11, 12). Communication with anesthesia for reverse Tendelenburg positioning, controlled hypotension, and pre-operative delivery of tranexamic acid also contributes to improve endoscopic visualization intra-operatively (11–14).

### Surgical technique

**Figure 2:**
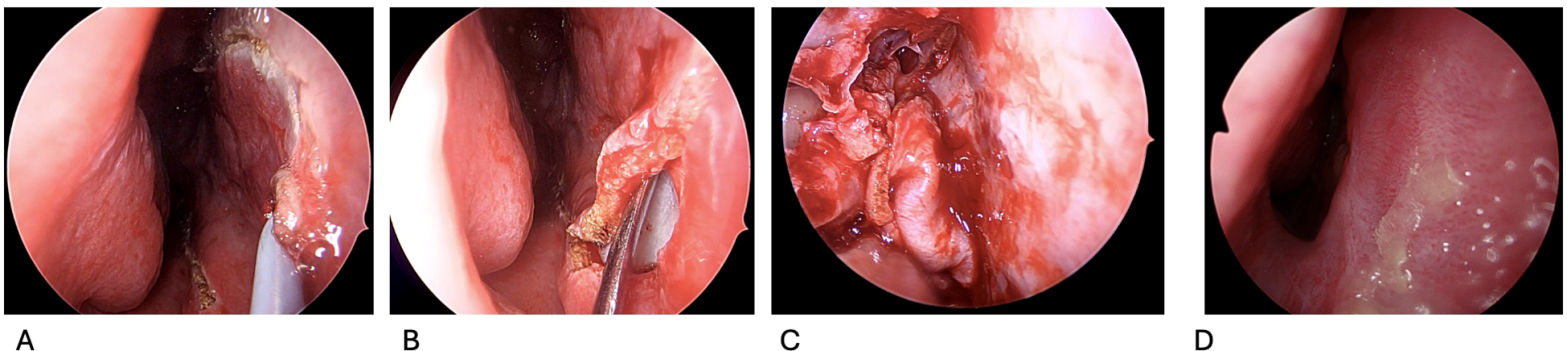
**(A–D)**. Endoscopic operative view right nasal cavity demonstrating incisions for flap harvest **(A)**, plane of flap harvest **(B)**, harvested flap and donor site **(C)**, and healed donor site post-operatively **(D**).

Surgical technique for the nasoseptal flap is achieved by raising a mucoperichondrial flap along the nasal septum without disturbing the posterior septal vascular pedicle or the olfactory mucosa (9). **Figures 1**, **2** illustrate the incisions for the flap in a schematic and the endoscopic intraoperative view, respectively. Endoscopic visualization is crucial to successful flap harvest, this can be optimized with topical decongestants, out fracturing the middle and inferior turbinates, controlled hypotension, systemic tranexamic acid and reverse Trendelenburg positioning (11–14). Resection of the middle turbinate will greatly improve visualization but must be balanced with the potential morbidity of turbinate resection.

The flap harvest begins once the sphenoid os has been identified. Two parallel incisions are made inferior to the os: the superior incision is immediately below the os and travels toward the nasal septum. It then curves sharply superiorly and runs along the superior aspect of the nasal septum (9). Care should be taken to remain 1–2 cm below the superior-most aspect of the septum to respect and avoid disrupting olfactory epithelium (9). The inferior incision classically starts along the top of the choana and heads toward the septum. It then turns abruptly inferiorly and runs along the floor of the nose over the maxillary crest (9). It should be noted that this inferior incision can extend onto the floor of the nasal cavity, or even further laterally onto the lateral nasal wall, to further increase the width of the flap (15–19). The two incisions are connected anteriorly with a vertical incision.

While respecting and maintaining as much mucosal coverage as possible is important to ensure paranasal sinus function post-operatively, it is generally advised to harvest a flap slightly larger than the expected defect to allow for a margin of error (9). As mentioned, this can be facilitated by taking the lower cut down onto the floor of the nose or the lateral nasal wall. The initial incisions can be made with cold steel instruments or a needle tip monopolar cautery; adding a 90-degree bend to the needle-tip cautery can be advantageous.

The flap is categorized as a mucoperichondrial flap; therefore, the plane of elevation is just above the cartilage and below the perichondrium, akin to raising a mucoperichondrial flap for a septoplasty (9). Flap elevation can be achieved using a freer or Cottle elevator. The use of suction elevation instruments may aid with visualization. The flap can then be rotated into the defect. Proper orientation of the flap is important to avoid kinking the pedicle and ensuring correct orientation of the mucosal surface. Modifications to increase the area of harvest have been described by extending the incisions and area of elevation laterally along the floor of the nose onto the inferior turbinate and lateral nasal wall (15–19).

The nasoseptal flap is generally used for repair of CSF leak, most commonly after transsphenoidal pituitary surgery. However, not all transsphenoidal cases will necessarily result in a CSF leak, making flap harvest unnecessary at times. In response to this, the concept of the ‘rescue flap’ has been developed (20). This technique includes making the superior cut and raising just the superior and posterior aspect of the flap to protect the pedicle during sphenoid surgery (20). If a CSF leak is encountered, then the flap is fully raised and utilized (20). However, if no CSF leak is encountered, the flap is laid back down on the nasal septum, effectively reducing operative time and septal mucosa in these scenarios (20). This can dramatically reduce the amount of exposed cartilage and results is less crusting post-operatively.

Multi-layer closure of skull base defects is often utilized, especially with high-flow CSF leaks (9, 21) but it should be noted that this will be practitioner dependent and often depends on if the leak is felt to be high-volume or low-volume. Free fat from the abdomen or thigh, as well as fascia, can be utilized to help seal off the leak, which can then be covered by a nasoseptal flap (9, 21). The flap can then be secured using biologic glues and tissue sealant (9, 21), with Tisseel or DuraSeal being options (22). While tissue glues are frequently used to secure the flap, their benefit in reducing CSF rhinorrhea is questionable (23). Surgifoam or Surgicel may also be used to bolster the flap. Some authors use Foley catheters, tampon sponges or other devices to bolster the site 5–7 days post-operatively (9, 24). Silastic sheets may also be placed to protect the raw, exposed donor site and promote healing post-operatively (9, 24).

### Post-operative care

Post-operatively, reducing intracranial pressure promotes successful reconstruction. Scheduled stool softeners, keeping head elevated and avoiding nose blowing or activities that will raise intracranial pressure are crucial (9, 11, 24). Nasal saline spray is often used post-operatively with nasal saline lavage commencing anywhere from 0–4 weeks post-operatively (9, 11, 24). Silastic sheets are typically removed 1–4 weeks post-operatively (9, 11, 24). Nasal debridement in clinic may be performed as well.

The use of silastic sheets has been debated, balancing patient discomfort with ideal healing. The consensus leans toward the use of silastic sheets with as they are shown to reduce meatal adhesions in endoscopic sinus surgery and have good patient tolerance (25, 26). The timing of splint removal is also noted to vary from 1–4 weeks post-operatively. At our institution, we lean toward a longer duration of sheets remaining intranasally to promote healing. However, future literature may aim to characterize the ideal timing of silastic sheet removal post-nasoseptal flap surgery and whether there is a time threshold where benefits are capped. A randomized control trial in septoplasty patients showed limited difference in the three-day compared to seven-day duration of silastic sheets post-operatively (27).

To further promote healing, some authors have suggested the use of free mucosal/fascia latae grafts or a reverse rotation graft to cover the donor site (28–30). However, evidence is limited beyond the use of silastics sheets and would require more robust analysis to conclude benefits.

Controversy also persisted surrounding the type and duration of antibiotic prophylaxis in endonasal skull base surgery, given the inherent risk of meningitis when exposing intracranial contents to the nasal cavity. A 2023 meta-analysis revealed that the use of multiple antibiotics and/or longer duration of antibiotics did not reduce the risk of post-operative infection (31). At our institution, ceftriaxone with dosing to penetrate the central nervous system is utilized peri-operatively.

Historically, lumbar drains were considered to reduce CSF pressure on the flap reconstruction; however, this is falling out of favor in routine cases given the risks of tension pneumocephalus, intracranial hypotension and the inherent risks of lumbar drain placement (9, 32).

### Complications

As with any tissue transfer, flap necrosis/failure is possible, but rates are extremely low (<1.3%) (15, 23, 33, 34). Meticulous intra-operative dissection, flap planning, and care to not disrupt the pedicle during harvest or kink the pedicle during inset can reduce the risk of flap necrosis (15). Identifying the sphenoid os early and then raising the flap before enlargement of the sphenoidotomy, is essential to ensure capture of the posterior septal branch of the SPA. Management post-operatively involves early recognition, debridement, irrigations, antibiotics and consideration of re-reconstruction if there is residual CSF leak or evidence of infection (15).

Mucocele formation occurs in < 4% of patients and is due to persistent mucosa on the recipient bed (33, 35). Complete stripping of the mucosa before reconstruction can prevent mucocele formation.

Patients may experience olfactory dysfunction post-nasoseptal flap. Upadhyay et al., 2017 noted lower University of Pennsylvania Smell Identification Test (UPSIT) scores with nasoseptal flap reconstruction compared to free mucosal grafts at 6 weeks, but this difference was no longer present at 3 months (29). Counseling patients pre-operatively that temporary hyposmia is expected may help them understand the post-operative course. Further, reducing trauma and dissection superiorly along the nasal septum aids in preserving the olfactory epithelium (9, 15, 33, 36).

In 2010, de Almeida et al. found that 98% of patients experienced nasal crusting post-endoscopic skull base surgery (30). Nasal crusting and dryness can be treated post-operatively with saline irrigations, humidification and intermittent debridement (15, 33). As well, silastic sheets along the septum are theorized to reduce crusting by protecting the raw surface of the septum. de Almeida et al. also found that the median time to re-mucosalization after nasoseptal flap was 89.0 days and the median time to absence of crusting was 101.0 days (30).

Septal perforation is reported in 0.9-14.4% (15, 33, 35, 37, 38). Careful handling of tissues intra-operatively while ensuring cartilage and contralateral mucosa and perichondrium remain intact may minimize the incidence of post-operative septal perforation.

Lastly, residual post-operative CSF leak occurs in < 5% of cases (8, 9, 15, 33). Small leaks may be managed conservatively with rest and head of bed elevation; high flow or persistent leaks often need to be treated with a return to the operating room for revision surgery (6, 15).

## Limitations of existing data & application to sinonasal malignancies

The use of the nasoseptal flap garnered popularity through its evidence in reducing CSF leaks in transsphenoidal pituitary surgery, and this is highlighted above. However, it’s use is also popularized in reconstruction following endoscopic resection of sinonasal malignancies, especially when resection results in a CSF leak. Despite this, limited patient series and cohorts exist looking at outcomes of nasoseptal flap reconstruction for sinonasal malignancies. Future literature may aim to compare outcomes following nasoseptal flap reconstruction for malignant and benign pathology.

More literature on outcomes following the use of the nasoseptal flap, not solely for transsphenoidal surgery, may also allow for better understanding of the versatility of this technique. Literature has demonstrated its use for repairing orbital floor defects in the maxillary sinus, palatal defects, and oropharyngeal reconstruction following transoral robotic surgery (39–42).

Further, in malignant cases, the use of radiotherapy may be necessary, and literature has shown increased risk of flap necrosis and poorer quality of life outcomes following nasoseptal flap reconstruction in patients who undergo radiotherapy (43, 44). Further literature on long-term patient outcomes, quality of life and factors associated with improved flap outcomes would strengthen our current understanding.

## Other locoregional reconstruction

While the nasoseptal flap is effective for endonasal reconstruction, malignancies involving the nasal septum may sacrifice the ability to use the nasoseptal flap. Inferior turbinate and lateral nasal wall flaps pedicled on the inferior turbinate artery have been reported as alternative endonasal vascularized reconstructive options for sinonasal malignancies (45, 46). Further, the pericranial flap can be harvested from an extra-nasal approach, typically involving a bi-coronal approach for harvest, and it is then tunnelled intra-nasally to reconstruct the skull base endoscopically (45, 47).

## Conclusion

Sinonasal malignancies can leave complex defects that can impact patients’ health and quality of life. Robust reconstruction of these defects is paramount to avoid devastating complications such as pneumocephalus and meningitis; this must be balanced with maintaining paranasal function and the patient’s quality of life. ESS has revolutionized the field of sinonasal malignancy resection and reconstruction. At the forefront of purely endoscopic reconstruction is the nasoseptal flap which has become the cornerstone of anterior skull base reconstruction. The nasoseptal flap and adjunctive reconstruction are discussed in depth in this narrative review.

## References

[1] Dutta R Dubal PM Svider PF Liu JK Baredes S Eloy JA . Sinonasal Malignancies: A population-based analysis of site-specific incidence and survival. Laryngoscope. (2015) 125:2491–7. doi: 10.1002/lary.25465, PMID: 26228792

[2] Thawani R Kim MS Arastu A Feng Z West MT Taflin NF . The contemporary management of cancers of the sinonasal tract in adults. CA Cancer J Clin. (2023) 73:72–112. doi: 10.3322/caac.21752, PMID: 35916666 PMC9840681

[3] Mauthe T Holzmann D Soyka MB Mueller SA Balermpas P Held U . Overall and disease-specific survival of sinonasal adenoid cystic carcinoma: a systematic review and meta-analysis. Rhinology. (2023) 61:508–18. doi: 10.4193/Rhin23.204, PMID: 37703531

[4] Bracigliano A Tatangelo F Perri F Lorenzo GD Tafuto R Ottaiano A . Malignant sinonasal tumors: update on histological and clinical management. Curr Oncol. (2021) 28:2420–38. doi: 10.3390/curroncol28040222, PMID: 34287240 PMC8293118

[5] de Almeida JR Hueniken K Xie M Monteiro E Zadeh G Kalyvas A . Multi-institutional comparison of quality of life between open versus endoscopic skull base approaches. Laryngoscope Investig Otolaryngol. (2025) 10:e70082. doi: 10.1002/lio2.70082, PMID: 39840025 PMC11748208

[6] Liu JK Das K Weiss MH Laws ER Jr Couldwell WT . The history and evolution of transsphenoidal surgery. J Neurosurg. (2001) 95:1083–96. doi: 10.3171/jns.2001.95.6.1083, PMID: 11765830

[7] Werner MT Yeoh D Fastenberg JH Chaskes MB Pollack AZ Boockvar JA . Reconstruction of the anterior skull base using the nasoseptal flap: A review. Cancers (Basel). (2023) 16:169. doi: 10.3390/cancers16010169, PMID: 38201596 PMC10778443

[8] Horridge M Jesurasa A Olubajo F Mirza S Sinha S . The use of the nasoseptal flap to reduce the rate of post-operative cerebrospinal fluid leaks following endoscopic trans-sphenoidal surgery for pituitary disease. Br J Neurosurgery. (2013) 27:739–41. doi: 10.3109/02688697.2013.795525, PMID: 23692070

[9] Hadad G Bassagasteguy L Carrau RL Mataza JC Kassam A Snyderman CH . A novel reconstructive technique after endoscopic expanded endonasal approaches: vascular pedicle nasoseptal flap. Laryngoscope. (2006) 116:1882–6. doi: 10.1097/01.mlg.0000234933.37779.e4, PMID: 17003708

[10] Pinheiro-Neto CD Snyderman CH . Nasoseptal flap. Adv Otorhinolaryngol. (2013) 74:42–55. doi: 10.1159/000342271, PMID: 23257551

[11] Liu JK Schmidt RF Choudhry OJ Shukla PA Eloy JA . Surgical nuances for nasoseptal flap reconstruction of cranial base defects with high-flow cerebrospinal fluid leaks after endoscopic skull base surgery. Neurosurg Focus. (2012) 32:E7. doi: 10.3171/2012.5.FOCUS1255, PMID: 22655696

[12] Khanwalkar AR Welch KC . Updates in techniques for improved visualization in sinus surgery. Curr Opin Otolaryngol Head Neck Surg. (2021) 29:9–20. doi: 10.1097/MOO.0000000000000693, PMID: 33315617

[13] Yang W Gou H Li H Liu Y Wan Y Wang C . Intravenous tranexamic acid improves the intraoperative visualization of endoscopic sinus surgery for high-grade chronic rhinosinusitis: A randomized, controlled, double-blinded prospective trial. Front Surg. (2021) 8:771159. doi: 10.3389/fsurg.2021.771159, PMID: 34869568 PMC8635022

[14] Zhang K Wang L Qi F Meng T . Hypotensive levels on endoscopic sinus surgery visibility: A randomized non-inferiority trial. Laryngoscope. (2024) 134:569–76. doi: 10.1002/lary.30867, PMID: 37449719

[15] Tang SH Hoerter JE Kshirsagar RS . History of and modern uses for the nasoseptal flap in skull base reconstruction after sinonasal Malignancy. Operative Techniques Otolaryngology-Head Neck Surgery. (2025) 36:4:308–318. doi: 10.1016/j.otot.2025.05.001, PMID: 41771284

[16] Zanation AM Carrau RL Snyderman CH Germanwala AV Gardner PA Prevedello DM . Nasoseptal flap reconstruction of high flow intraoperative cerebral spinal fluid leaks during endoscopic skull base surgery. Am J Rhinol Allergy. (2009) 23:518–21. doi: 10.2500/ajra.2009.23.3378, PMID: 19807986

[17] Peris-Celda M Pinheiro-Neto CD Funaki T Fernandez-Miranda JC Gardner P Snyderman C . The extended nasoseptal flap for skull base reconstruction of the clival region: an anatomical and radiological study. J Neurol Surg B Skull Base. (2013) 74:369–85. doi: 10.1055/s-0033-1347368, PMID: 24436940 PMC3836807

[18] Moon JH Kim EH Kim SH . Various modifications of a vascularized nasoseptal flap for repair of extensive skull base dural defects. J Neurosurg JNS. (2020) 132:371–9. doi: 10.3171/2018.10.JNS181556, PMID: 30738381

[19] Boetto J Labidi M Watanabe K Hanakita S Bouazza S Passeri T . Combined nasoseptal and inferior turbinate flap for reconstruction of large skull base defect after expanded endonasal approach: operative technique. Oper Neurosurg. (2019) 16:45–52. doi: 10.1093/ons/opy046, PMID: 29617919

[20] Rivera-Serrano CM Snyderman CH Gardner P Prevedello D Wheless S Kassam AB . Nasoseptal “rescue” flap: a novel modification of the nasoseptal flap technique for pituitary surgery. Laryngoscope. (2011) 121:990–3. doi: 10.1002/lary.21419, PMID: 21520113

[21] Kim BK Kong DS Nam DH Hong SD . Comparison of graft materials in multilayer reconstruction with nasoseptal flap for high-flow CSF leak during endoscopic skull base surgery. J Clin Med. (2022) 11:6711. doi: 10.3390/jcm11226711, PMID: 36431187 PMC9697000

[22] Chin CJ Kus L Rotenberg BW . Use of duraseal in repair of cerebrospinal fluid leaks. J Otolaryngol Head Neck Surg. (2010) 39:594–9. 20828525

[23] Chabot JD Patel CR Hughes MA Wang EW Snyderman CH Gardner PA . Nasoseptal flap necrosis: a rare complication of endoscopic endonasal surgery. J Neurosurg. (2018) 128:1463–72. doi: 10.3171/2017.2.JNS161582, PMID: 28731395

[24] Wheless SA McKinney KA Carrau RL Snyderman CH Kassam AB Germanwala AV . Nasoseptal flap closure of traumatic cerebrospinal fluid leaks. Skull Base. (2011) 21:93–8. doi: 10.1055/s-0030-1266763, PMID: 22451808 PMC3312593

[25] Baguley CJ Stow NW Weitzel EK Douglas RG . Silastic splints reduce middle meatal adhesions after endoscopic sinus surgery. Am J Rhinol Allergy. (2012) 26:414–7. doi: 10.2500/ajra.2012.26.3810, PMID: 23168159

[26] Wadhera R Zafar N Gulati SP Kalra V Ghai A . Comparative study of intranasal septal splints and nasal packs in patients undergoing nasal septal surgery. Ear Nose Throat J. (2014) 93:396–408. 25255346

[27] Arslan S Yıldırım H Çobanoğlu B Işık AÜ Bahadır O . Impact of intranasal splint removal time on postoperative complications after septoplasty. Niger J Clin Pract. (2024) 27:430–4. doi: 10.4103/njcp.njcp_381_23, PMID: 38679763

[28] Kimple AJ Leight WD Wheless SA Zanation AM . Reducing nasal morbidity after skull base reconstruction with the nasoseptal flap: free middle turbinate mucosal grafts. Laryngoscope. (2012) 122:1920–4. doi: 10.1002/lary.23325, PMID: 22926937 PMC3457916

[29] Zeinalizadeh M Sadrehosseini SM Barkhoudarian G Carrau RL . Reconstruction of the denuded nasoseptal flap donor site with a free fascia lata graft: technical note. Eur Arch Otorhinolaryngol. (2016) 273:3179–82. doi: 10.1007/s00405-016-3962-0, PMID: 26951218

[30] Caicedo-Granados E Carrau R Snyderman CH Prevedello D Fernandez-Miranda J Gardner P . Reverse rotation flap for reconstruction of donor site after vascular pedicled nasoseptal flap in skull base surgery. Laryngoscope. (2010) 120:1550–2. doi: 10.1002/lary.20975, PMID: 20564666

[31] Kwon TH Shin HK Yoon WK Kim JH Byun J . Antibiotics prophylaxis for endoscopic endonasal approach for skull base tumor surgery: A meta-analysis. World Neurosurg. (2023) 174:e82–91. doi: 10.1016/j.wneu.2023.02.143, PMID: 36894007

[32] Eloy JA Kuperan AB Choudhry OJ Harirchian S Liu JK . Efficacy of the pedicled nasoseptal flap without cerebrospinal fluid (CSF) diversion for repair of skull base defects: incidence of postoperative CSF leaks. Int Forum Allergy Rhinol. (2012) 2:397–401. doi: 10.1002/alr.21040, PMID: 22528686

[33] Lavigne P Faden DL Wang EW Snyderman CH . Complications of nasoseptal flap reconstruction: A systematic review. J Neurol Surg B Skull Base. (2018) 79:S291–9. doi: 10.1055/s-0038-1668158, PMID: 30210981 PMC6133677

[34] Yamada H Toda M Fukumura M Imai R Ozawa H Ogawa K . Cerebrospinal fluid leakage due to nasoseptal flap partial necrosis: A pitfall for skull base reconstruction of endoscopic endonasal surgery. Surg Neurol Int. (2020) 11:121. doi: 10.25259/SNI_117_2020, PMID: 32494396 PMC7265470

[35] Soudry E Psaltis AJ Lee KH Vaezafshar R Nayak JV Hwang PH . Complications associated with the pedicled nasoseptal flap for skull base reconstruction. Laryngoscope. (2015) 125:80–5. doi: 10.1002/lary.24863, PMID: 25111727

[36] Upadhyay S Buohliqah L Dolci RLL Otto BA Prevedello DM Carrau RL . Periodic olfactory assessment in patients undergoing skull base surgery with preservation of the olfactory strip. Laryngoscope. (2017) 127:1970–5. doi: 10.1002/lary.26546, PMID: 28349579

[37] de Almeida JR Snyderman CH Gardner PA Carrau RL Vescan AD . Nasal morbidity following endoscopic skull base surgery: a prospective cohort study. Head Neck. (2011) 33:547–51. doi: 10.1002/hed.21483, PMID: 20824807

[38] Rowan NR Wang EW Gardner PA Fernandez-Miranda JC Snyderman CH . Nasal deformities following nasoseptal flap reconstruction of skull base defects. J Neurol Surg B Skull Base. (2016) 77:14–8. doi: 10.1055/s-0035-1555136, PMID: 26949583 PMC4777617

[39] Kalyoussef E Schmidt RF Liu JK Eloy JA . Structural pedicled mucochondral-osteal nasoseptal flap: a novel method for orbital floor reconstruction after sinonasal and skull base tumor resection. Int Forum Allergy Rhinol. (2014) 4:577–82. doi: 10.1002/alr.21306, PMID: 24574271

[40] Gadkaree SK Feng AL Sharon JD Richmond JD Emerick KS Lin DT . Composite nasoseptal flap for palate reconstruction. J Craniofac Surg. (2019) 30:1990–3. doi: 10.1097/SCS.0000000000005652, PMID: 31205277

[41] Alwashahi MK Battaglia P Turri-Zanoni M Castelnuovo P . Nasoseptal flap for palatal reconstruction after hemi-maxillectomy: case report. J Laryngol Otol. (2018) 132:83–7. doi: 10.1017/S002221511700233X, PMID: 29151373

[42] Pinheiro-Neto CD Galati LT . Nasoseptal flap for reconstruction after robotic radical tonsillectomy. Head Neck. (2016) 38:E2495–8. doi: 10.1002/hed.24483, PMID: 27142938

[43] Gong Q Li H Liu H Shi Y . Multifactorial clinical analysis of factors affecting necrosis of nasal septal mucosal flap after salvage surgery for recurrent nasopharyngeal carcinoma. Sci Rep. (2024) 14:29287. doi: 10.1038/s41598-024-80800-9, PMID: 39592834 PMC11599838

[44] Shay A Sturgis M Ritz EM Beer-Furlan A Munoz L Byrne R . Prior smoking and nasoseptal flap usage adversely impact quality of life and healing after endoscopic pituitary surgery. Neurosurg Focus. (2020) 48:E17. doi: 10.3171/2020.3.FOCUS2050, PMID: 32480369

[45] Kraimer K Geltzeiler M . Skull base reconstruction by subsite after sinonasal Malignancy resection. Cancers (Basel). (2024) 16:242. doi: 10.3390/cancers16020242, PMID: 38254733 PMC10813932

[46] Patel MR Taylor RJ Hackman TG Germanwala AV Sasaki-Adams D Ewend MG . Beyond the nasoseptal flap: outcomes and pearls with secondary flaps in endoscopic endonasal skull base reconstruction. Laryngoscope. (2014) 124:846–52. doi: 10.1002/lary.24319, PMID: 23877996

[47] Gil Z Abergel A Leider-Trejo L Khafif A Margalit N Amir A . A comprehensive algorithm for anterior skull base reconstruction after oncological resections. Skull Base. (2007) 17:25–37. doi: 10.1055/s-2006-959333, PMID: 17603642 PMC1852574

